# Mushroom management style as a predictor of teachers’ quiet quitting behavior

**DOI:** 10.1186/s40359-026-05447-9

**Published:** 2026-09-01

**Authors:** Erdal Meriç

**Affiliations:** Ordu University, Fatsa Vocational School, Ordu, Türkiye

**Keywords:** Educational management, School administration, Mushroom management, Quiet quitting, Teacher

## Abstract

**Background:**

The mushroom management style refers to a management approach where employees’ access to information is restricted and they are not included in decision-making processes. Quiet quitting, conversely, it refers to employees only performing their mandatory duties without resigning, avoiding putting extra effort into their work. This study aims to investigate the extent to which mushroom management style predicts quiet quitting behavior among public school teachers from the perspective of Social Exchange Theory and Psychological Contract Theory.

**Methods:**

This quantitative study is designed using a correlational survey model. The study group consists of 460 teachers selected using simple random sampling across seven geographical regions of Türkiye. Data were gathered through the “Mushroom Management Scale” and the “Teachers’ Quiet Quitting Scale,” and correlation and regression techniques were used in the analyses.

**Results:**

The results indicate that teachers’ perceptions of mushroom management style were generally low, while their perceptions were moderate in terms of information sharing and communication. Quiet quitting behaviors were generally very low, but were also found to be at a low level in terms of depersonalization.

**Conclusions:**

The findings indicated a moderate, positive, and statistically significant relationship between mushroom management style and quiet quitting behavior. In addition, mushroom management emerged as a significant predictor of quiet quitting behavior. In light of these results, several implications for practice and directions for future research are proposed.

## Introduction

One of the significant challenges organizations face in modern working life is the emergence of negative work behaviors stemming from suboptimal managerial practices [1]. A distinctly detrimental approach that has recently gained attention in the management literature is the “mushroom management” style. This concept refers to a covert control tactic where employees’ access to information is intentionally restricted, they are excluded from decision-making processes, and they are g enerally “kept in the dark,” expected merely to perform assigned tasks without feedback [2]. Although the literature shows that this type of lack of administrative transparency negatively impacts employee performance and consequently increases intention to leave the job [3–5], its role in passive organizational withdrawal has not been sufficiently investigated.

A reflection of this withdrawal is “quiet quitting,” a behavioral pattern in which employees limit their efforts to only the tasks they are obligated to perform and do not offer the extra contribution that would benefit their organization [6]. While quiet quitting has been linked to broader organizational dysfunctions such as burnout, alienation, and cynicism [7–9], Zenger and Folkman [10] note that a more in-depth empirical investigation into its direct link to specific toxic leadership styles is needed. Although the literature addresses the general consequences of top-down management failures, there is a significant empirical gap regarding how intentional information asymmetry—particularly “mushroom management”—contributes to quiet quitting in educational settings. Therefore, this study aims to investigate, from the perspectives of Social Change Theory and Psychological Contract Theory, the extent to which the “mushroom management” style predicts “quiet quitting” behavior among public school teachers.

Teaching is a profession based on high motivation and voluntary contributions outside of job descriptions [11]; institutional success is also related to these efforts. The lack of transparency in mushroom management and its exclusion of teachers from decision-making processes is a situation rooted in the psychological contract between the institution and its employees. Social Change Theory evaluates the decrease in organizational commitment and effort levels of employees who do not receive transparent support from management in this context [12]. While the effects of managerial toxicity are known, empirical data on the relationship between quiet quitting and mushroom management style among teachers are limited. This study examines the lack of managerial transparency and employee withdrawal behaviors together, focusing on the factors that maintain professional commitment in educational institutions and the value of transparent practices.

## Theoretical framework

### Mushroom management

The mushroom management style is an approach where information sharing with employees is restricted, and they are only given a minimal amount of information necessary to perform their jobs [13]. Based on the metaphor of mushroom cultivation, this management style symbolizes a structure where employees are “kept in the dark,” fed only with instructions instead of feedback, and yet high productivity is expected of them [14]. In the literature, this management approach is widely considered a negative management practice, particularly because of its lack of participation and transparency, its weakening of superior-subordinate relationships and consequently its leading to power intoxication among superiors, its limitation of employees’ ability to develop innovative ideas and use problem-solving skills, and its weakening of organizational efficiency and competitiveness [15]. However, a minority view argues that it has positive contributions such as protecting confidentiality within the organization, preventing crises, and consequently reducing organizational chaos and employee stress [16].

To further clarify the mushroom management concept, related concepts such as authoritarian leadership, communication climate, and organizational silence need to be clarified.

While authoritarian leadership describes a management style where dominance and all power are concentrated in a single person [17], mushroom management, as a more covert control mechanism, essentially relies on a deliberate lack of transparency and information asymmetry.

A weak communication climate often stems from structural inefficiencies [18]. In contrast, mushroom management is a deliberate leadership practice characterized by the strategic hoarding of information to maintain managerial power. While the communication climate concerns the overall quality of communication within an organization, mushroom management focuses on the deliberate information restriction imposed by leaders. This distinction allows this study to highlight the role of deliberate information control as a defining characteristic of mushroom management.

Mushroom management and organizational silence are both harmful to organizations, but they originate from different sources. Mushroom management is a conscious, top-down strategy employed to consolidate power through the deliberate withholding of information. Organizational silence, on the other hand, typically arises from the bottom up; it is a defensive response based on an employee’s belief that speaking up is either futile or professionally dangerous [19]. One is the imposition of structural ambiguity, while the other is a defensive self-protection measure employed by individuals in an environment that discourages openness.

Mushroom management, through the deliberate restriction of information, inevitably shapes employee reactions and bottom-up responses. A synthesis of existing studies shows that when leadership consistently excludes employees from decision-making processes and fails to provide consistent justifications, it leads to the spread of organizational cynicism [20, 21]. Furthermore, rather than viewing organizational silence as merely a parallel problem, it is more accurate to conceptualize it as a withdrawal behavior at the employee level; this is a reactive response triggered by the lack of top-down transparency inherent in mushroom management [22–25].

While silence and cynicism are known consequences of inadequate communication, it has not yet been proven whether school administration’s deliberate withholding of information leads to quiet quitting. Since the teaching profession relies heavily on voluntary effort, examining this particular link is practically important. Therefore, this study aims to determine whether a lack of administrative transparency plays a measurable role in teachers’ decisions to reduce their efforts.

### Quiet quitting

The concept of quiet quitting emerged to describe a behavioral pattern where employees strictly adhere to their mandatory job descriptions but consciously minimize extra-role contributions and organizational citizenship behaviors [10, 26, 27]. Researchers in the literature approach this phenomenon from somewhat different perspectives. While some researchers frame quiet quitting primarily as a boundary-setting mechanism focused on protecting employee well-being [28], a broader consensus conceptualizes it as a strong indicator of weakened psychological commitment and decreased organizational fit [29, 30]. Unlike those who formally resign, quiet quitters although still effectively in their positions, mentally distance themselves from efforts that benefit the organization [11]. Consequently, the defining characteristic of quiet quitting is its implicit nature; it involves underlying dynamics of psychological withdrawal that are not easily detectable from the outside [31–33].

When examining the causes of quiet quitting, studies generally differentiate between macro-environmental factors and specific managerial failures. Some researchers attribute this withdrawal to issues such as excessive working hours, digitalization-induced burnout, or general work-workplace mismatch [34]. Others suggest that quiet quitting is largely a reaction to poor leadership and feelings of being undervalued. Employees who feel undervalued or lack external motivation tend to reduce their commitment and quiet quitting as a reaction to their employers’ actions [10, 35–37].

While the literature addresses the relationships between general managerial neglect and quiet quitting, the specific role of deliberate information withholding has not been sufficiently investigated. Research links employee withdrawal to perceived lack of appreciation or poor communication, but the significant impact of mushroom management, characterized by deliberate information asymmetry, has not been isolated in this context. While the effects of overtly toxic leadership are well known, how covert managerial tactics like mushroom management shape passive withdrawal of teachers remains unclear. Given the voluntary and merit-based nature of the teaching profession, examining this theoretical gap is considered important.

### Social exchange theory

Social Exchange Theory is known as a rational approach that defines social behavior as outcome-oriented, basing individuals’ social interactions within a group on a cost-benefit balance [38]. Blau [39] emphasizes not only the economic dimension of this approach but also its structure that develops through voluntary actions and trust, where the principle of reciprocity emerges. According to this principle, employees feel obligated to reciprocate what they receive from their organizations [40]. In Social Exchange Theory, appreciation and the recognition and evaluation of success are paramount. In this context, when employees believe they are receiving sufficient support from the organization, they will exhibit higher commitment, job satisfaction, and organizational citizenship behavior based on the principle of reciprocity [41]. According to Social Exchange Theory, employees who believe that they do not receive adequate organizational support may perceive a psychological contract breach and exhibit quiet quitting behavior [12, 40]. Therefore, it can be said that employees’ perceptions of whether their organizations value them shape the content of the psychological contract.

While it is known that a lack of organizational support reduces citizenship behavior, the impact of deliberate information withholding on this process has not been sufficiently studied. While the literature attributes employee withdrawal behavior to general negativity or mistreatment, limited attention has been paid to the social change distortions created by mushroom management. In particular, whether teachers resort to quiet quitting to establish reciprocity in situations of “kept in the dark” is an area that needs clarification.

### Psychological contract theory

Psychological Contract Theory emphasizes the implicit expectations mutually held by employees and organizations, representing the sum of unwritten obligations of mutual commitment between the organization and its employees based on trust, loyalty, and fairness [42]. It is known that breaches of this contract by the organization can lead to serious negative consequences for employees, including decreased performance, alienation from work, quiet quitting, and even intention to leave the job. The principle of reciprocity is also present in this theory, based entirely on the feeling of obligation to reciprocate kindness with kindness [43]. Similarly, the quality of the relationship between leader and employee is also an important contextual variable determining the perception of the psychological contract [44].

Despite the clear link between psychological contract breaches and negative employee attitudes, the specific impact of intentional information asymmetry has received limited attention. While poor leadership often damages the psychological contract, empirical research has not sufficiently addressed how the intentional withholding of information in mushroom management is interpreted as a breach or how it relates to quiet quitting. The role of information restriction as a passive withdrawal mechanism has not been adequately explored, particularly in educational settings. Therefore, this study aims to investigate whether mushroom management contributes to quiet quitting by acting as a type of psychological contract breach.

### Mushroom management style and quiet quitting behavior from the perspective of social exchange theory and psychological contract theory

Approaches such as Social Exchange Theory and Psychological Contract Theory are among the fundamental theoretical frameworks widely used to explain employee attitudes and behaviors [39, 45]. However, the effectiveness of these theories in explaining the relationship between implicit and negative leadership practices such as “Mushroom Management” and new-generation employee behaviors such as “Quiet Quitting” has not yet been sufficiently empirically tested [46]. In this context, examining this relationship has the potential to significantly extend the literature by enabling existing theoretical approaches to be tested in a different organizational context.

In mushroom management style, information sharing is limited, employees are excluded from decision-making processes, and employee expectations are not met. Such a management style weakens employees’ commitment to the organization, undermines organizational trust, and leads to negative organizational behaviors. In today’s organizations, where leaving the job is not possible due to economic reasons, these negative organizational behaviors can manifest as quiet quitting [12]. From the perspective of Social Exchange Theory, this situation may be interpreted as a result of the weakening reciprocity within the employee–organization relationship.

Employees shape their performance based on the perceived level of support they receive from the organization, and may tend to limit their contributions when the balance of reciprocity is disrupted [39]. Within the context of Psychological Contract Theory, quiet quitting is considered an attitude towards perceived contract breaches; in this process, employees may choose to minimize their efforts and remain limited by their role descriptions rather than directly resigning when there is a mismatch between rewards and expectations [45, 47]. In cases of imbalance between high performance expectations and perceived value, quiet quitting is a reflection of organizational injustices and reciprocity violations rather than a lack of individual motivation [32]. This behavior is considered a passive strategy aimed at re-establishing the balance of reciprocity without completely terminating organizational commitment; this approach is consistent with theoretical frameworks that link employee behavior to the quality of organizational relationships.

Professional commitment relies on a transparent organizational environment where healthy communication exists. Management styles that lack transparency and restrict information sharing create an environment that contradicts the principle of reciprocity. This situation can be linked to teachers resorting to quiet quitting as a coping mechanism, limiting their voluntary efforts to maintain a cost-benefit balance. Testing this dynamic in educational settings can help clarify how opaque management directlyaffects withdrawal. Therefore, this research aims to investigate the relationship between the mushroom management style and teachers’ quiet quitting behaviors, thereby providing empirical data to the fields of educational management and organizational psychology.

## Methods

### Purpose of study

This study aims to investigate the extent to which mushroom management style predicts quiet quitting behavior among public school teachers from the perspective of Social Exchange Theory and Psychological Contract Theory. Consistent with this overall objective, the following research questions were examined.

1. What are the levels of teachers’ perceptions of mushroom management style and their quiet quitting behavior?
2. What is the association between teachers’ perceptions of mushroom management style and their quiet quitting behavior?
3. To what extent does mushroom management style predict quiet quitting behavior?

### Research design

This quantitative study is designed using a correlational survey model. A correlational survey model is intended to identify whether a relationship exists between two or more variables and, if so, to what extent they co-vary. This design was chosen because it allows for the evaluation of statistical relationships between naturally occurring organizational variables without experimental intervention [48].

### Study group

The study group comprised 460 teachers employed at 28 public schools selected via simple random sampling from seven provinces, each representing provinces across Türkiye’s seven geographical regions. These provinces were selected to account for geographical and socio-economic differences in the context of education. The study group compristed of 218 (47.4%) female and 242 (52.6%) male teachers, with 383 (83.3%) married and 77 (16.7%) single teachers. 380 (82.6%) of the teachers graduated from faculties of education, while 80 (17.4%) graduated from faculties other than education. Regarding educational background, 355 (77.2%) teachers held bachelor’s degrees, while 105 (22.8%) held postgraduate degrees. Looking at the educational levels at which they work, 76 (16.5%) teachers work in preschool, 136 (29.6%) in primary school, 127 (27.6%) in middle school, and 121 (26.3%) in high school. In terms of teaching experience, 256 teachers (55.7%) had 1–20 years of experience, while 204 teachers (44.3%) had 21 years or more. In terms of tenure at the current school, 226 teachers (49.1%) reported 1–5 years of service, whereas 234 teachers (50.9%) reported 6 years or more.

### Data collection instruments

This study utilized the following instruments for data collection.

#### Mushroom management scale (MMS)

Teachers’ perceptions of mushroom management were assessed using the “Mushroom Management Scale” constructed by Birincioğlu and Tekin [49]. The MMS, consisting of 19 items and four subscales, was named as “Inadequate information sharing” (6 items), “Fear of loss of power” (5 items), “Inadequate communication” (4 items), and “Lack of participative management” (4 items). The five-point Likert scale is ranges from “Never” (1) to “Always” (5). Higher scores reflect a greater perception of mushroom management among teachers. Items 4, 5, 9, 10, 12, and 14 on the scale are reverse coded.

The reliability of the MMS was assessed by Birincioğlu and Tekin [49] using data obtained from research assistants working in universities. The Cronbach’s alpha was calculated as 0.90 for the scale as a whole, and 0.90, 0.88, 0.81, and 0.84 for the subscales, respectively. A Confirmatory Factor Analysis (CFA) conducted with data from a different group of research assistants also confirmed that the scale’s four-dimensional structure was retained.

In this study, the Cronbach’s alpha coefficient for the overall scale was 0.93, while it ranged from 0.71 to 0.85 across the respective subscales. CFA was performed to assess the construct validity of the MMS, and the results indicated that item loadings ranged from 0.54 to 1.16, that the scale’s four-dimensional structure was retained, and that the overall model demonstrated a good to excellent level of fit (X^2^/df = 2.23, CFI = 0.96, NFI = 0.93, GFI = 0.93, AGFI = 0.90, TLI = 0.95, IFI = 0.96, RMSEA = 0.05, SRMR = 0.04, RMR = 0.05).

### Teachers’ quiet quitting scale (TQQS)

The quiet quitting behaviors of teachers were assessed using the “Teachers’ Quiet Quitting Scale” constructed by Yücedağlar et al. [50]. The TQQS, consisting of 17 items and three subscales, was named as “Work-related performance” (7 items), “Indifference towards school” (6 items), and “Work-related depersonalization” (4 items). The five-point Likert scale is ranges from “Never” (1) to “Always” (5). Higher scores reflect higher levels of quiet quitting behavior among teachers. Items 10 and 16 on the scale are reverse coded.

The reliability of the TQQS was assessed by Yücedağlar et al. [50] using data obtained from teacher participation, and the Cronbach’s alpha coefficients for the subscales of the scale were 0.85, 0.83, and 0.74, respectively. CFA was conducted on a different sample group to assess the construct validity of the scale, and the analysis confirmed that the scale’s three-dimensional structure was retained.

In this study, the Cronbach’s alpha coefficient for the overall scale was 0.88, while it ranged from 0.73 to 0.82 across the respective subscales. CFA was performed to assess the construct validity of the TQQS, and the results indicated that item loadings ranged from 0.82 to 1.33, that the scale’s three-dimensional structure was retained, and that the overall model demonstrated a good to excellent level of fit (X^2^/df = 2.47, CFI = 0.94, NFI = 0.90, GFI = 0.94, AGFI = 0.91, TLI = 0.92, IFI = 0.94, RMSEA = 0.05, SRMR = 0.04, RMR = 0.04).

### Data collection and analysis

Ethical approval for the research was obtained from the Ordu University Educational Research Ethics Committee (Decision No: 2026-111). The research data was collected electronically (Google Forms). The data obtained from scale forms completed voluntarily by 485 teachers were entered into the SPSS software for analysis. Prior to the main analyses, the dataset was screened for missing values and outliers. Because the survey instruments were configured to require complete responses for all focal items, missing data were negligible. For univariate outliers, 23 cases with z-scores exceeding ±3 were found to be outliers and excluded from the dataset. For multivariate outliers, Mahalanobis distance was initially examined, and two cases with p-values below 0.001 were found to be outliers and excluded from the dataset. Cook’s distance was also computed to examine potential outliers, and no cases with values exceeding 1.00 were identified. Therefore, the analyses were conducted with the remaining 460 cases.

Data analysis was conducted using SPSS, with statistical significance levels established at 0.01 and 0.05. Since the main objective of this study was to test directly predictive relationships and analyze variance through multiple linear regression, this software package was sufficient for the necessary analyses [51, 52]. Univariate normality was evaluated through an examination of skewness and kurtosis coefficients while scatterplot matrices were used to evaluate multivariate normality of the dataset. Univariate normality was assessed by calculating skewness and kurtosis coefficients for the total scores and subscales of both the MMS and TQQS. Regarding the MMS and its subscales, skewness coefficients were found to vary between 0.212 and 0.499, while kurtosis coefficients ranged from −0.883 to −0.507. In the case of the TQQS and its subscales, skewness coefficients were between 0.638 and 0.826, and kurtosis coefficients ranged from −0.669 to 0.245. According to Field [51], data are considered to be normally distributed when skewness and kurtosis coefficients fall within ±1. The analysis of skewness and kurtosis coefficients and scatterplots indicated that parametric tests were appropriate for the analyses.

Given that all variables in the study were were collected from the same respondents using self-report measures, additional statistical evidence should be provided to assess the extent of method bias. To assess common methodological bias, Harman’s Single-Factor Test was first applied. The first factor revealed by the unrotated exploratory factor analysis explained 30.639% of the total variance. Since this percentage is below the 50% threshold, it was concluded that common method bias does not pose a serious threat. Second, to provide more robust evidence, a single-factor confirmatory factor analysis in which all items were loaded onto a single latent factor was conducted. The analysis demonstrated a very poor fit to the data, with all indices falling well below acceptable thresholds (X²/df = 5.566, CFI = 0.64, NFI = 0.60, GFI = 0.58, AGFI = 0.53, TLI = 0.62, IFI = 0.64, RMSEA = 0.10, SRMR = 0.10, RMR = 0.10). Based on these two analyses, it was concluded that common methodological variance does not pose a serious threat to the validity of the findings.

This study, which aims to examine how mushroom management style predicts quiet quitting behavior, identified four subscales of mushroom management as predictor variables and quiet quitting behavior as the outcome variable. Multiple linear regression analysis was performed to investigate how mushroom management style predicts quiet quitting behavior. Prior to regression analysis, the model assumptions were assessed. The scatterplot matrix indicated that the data satisfied the assumption of multivariate normality and demonstrated linear associations between the predictor and outcome variables. In addition, no multicollinearity (VIF < 10; Tolerance > 0.10; CI < 30) or autocorrelation (1.5 < Durbin-Watson = 1.95 < 2.5) problems were detected among the predictor variables. The relationships among the variables were examined using Pearson correlation analysis. Additionally, mean and standard deviation (SD) coefficients were calculated as part of the data analysis. The scale scores were interpreted according to the following criteria: 1.00–1.79 (very low), 1.80–2.59 (low), 2.60–3.39 (moderate), 3.40–4.19 (high), and 4.20–5.00 (very high). Score intervals were established using a grouped scoring formula, and fixed cut-off coefficients were applied.

## Results

The results part first presents the mean and SD coefficients for teachers’ perceptions of mushroom management style and their levels of quiet quitting behavior, followed by results on the relationship between mushroom management style and quiet quitting behavior, and finally, the results of the multiple linear regression analysis examining the predictive effect of mushroom management style on quiet quitting behavior are presented.

### Teachers’ perceptions of mushroom management style and their levels of quiet quitting behavior

Table 1 presents the mean and SD coefficients regarding teachers’ perceptions of mushroom management style and their quiet quitting behavior levels.

**Table 1:** Teachers’ perceptions of mushroom management style and levels of quiet quitting behavior

Variables	n	M	SD
Mushroom Management Style	460	2.52	0.79
*Inadequate information sharing*	460	2.60	0.82
*Fear of loss of power*	460	2.25	0.94
*Inadequate communication*	460	2.72	0.91
*Lack of participative management*	460	2.53	1.03
Quiet Quitting Behavior	460	1.69	0.54
*Work-related performance*	460	1.46	0.51
*Indifference towards school*	460	1.75	0.66
*Work-related depersonalization*	460	2.00	0.87

As observed in Table 1, teachers’ overall perceptions of the mushroom management style, as well as their perceptions regarding the fear of loss of power and lack of participative management subscales, are at a low level, while their perceptions regarding the inadequate information sharing and inadequate communication subscales are at a medium level. Correspondingly, teachers’ overall quiet quitting behavior, as well as their behavior regarding the work-related performance and indifference towards school subscales, is at a very low level, while their behavior regarding the work-related depersonalization subscale is at a low level.

### Relationship between mushroom management style and quiet quitting behavior

Table 2 presents the results of the correlation analysis examining the relationship between teachers’ perceptions of mushroom management style and quiet quitting behavior.

**Table 2:** The relationship between mushroom management style and quiet quitting behavior

Variables	(1)	*(1.1)*	*(1.2)*	*(1.3)*	*(1.4)*	(2)	*(2.1)*	*(2.2)*	*(2.3)*
Mushroom Management Style (1)	1	0.889	0.914	0.761	0.873	0.449	0.342	0.488	0.265
*Inadequate information sharing (1.1)*	0.889	1	0.744	0.536	0.726	0.400	0.318	0.415	0.246
*Fear of loss of power (1.2)*	0.914	0.744	1	0.632	0.748	0.418	0.292	0.486	0.239
*Inadequate communication (1.3)*	0.761	0.536	0.632	1	0.529	0.323	0.248	0.345	0.196
*Lack of participative management (1.4)*	0.873	0.726	0.748	0.529	1	0.398	0.316	0.427	0.230
Quiet Quitting Behavior (2)	0.449	0.400	0.418	0.323	0.398	1	0.842	0.828	0.805
*Work-related performance (2.1)*	0.342	0.318	0.292	0.248	0.316	0.842	1	0.542	0.555
*Indifference towards school (2.2)*	0.488	0.415	0.486	0.345	0.427	0.828	0.542	1	0.467
*Work-related depersonalization (2.3)*	0.265	0.246	0.239	0.196	0.230	0.805	0.555	0.467	1

Table 2 indicates that there is a moderate, positive, and significant correlation between mushroom management style and quiet quitting behavior (*r* = 0.449, *p* < 0.01). Similarly, positive and significant correlations are observed between the subscales of mushroom management style and the subscales of quiet quitting behavior (0.196 ≤ *r* ≤ 0.486, *p* < 0.01).

### The predictive power of mushroom management style on quiet quitting behavior

The results of the multiple linear regression analysis examining the extent to which mushroom management style predicts quiet quitting behavior and its subscales are presented in the tables.

Table 3 presents the results of the multiple regression analysis examining the predictive effect of mushroom management style on quiet quitting behavior.

**Table 3:** Results of multiple regression analysis predicting quiet quitting behavior based on mushroom management style

Predictor Variables	Quiet Quitting Behavior						
	B	SE	β	t	p	Bivariate *r*	Partial *r*
Constant	0.949	0.083		11.402	0.00**		
Inadequate information sharing	0.090	0.044	0.138	2.034	0.04*	0.400	0.095
Fear of loss of power	0.099	0.043	0.173	2.303	0.02*	0.418	0.107
Inadequate communication	0.041	0.032	0.070	1.281	0.20	0.323	0.060
Lack of participative management	0.069	0.036	0.132	1.923	0.06	0.398	0.090

*R* = 0.450; *R*^*2*^ = 0.203; *F*_(4, 455)_ = 28.901; *p* < 0.01

As observed in Table 3, the overall regression model significantly accounts for approximately 20% of the variance in quiet quitting behavior (*R²* = 0.203, *F*_(4, 455)_ = 28.901, *p* < 0.01). Examination of the regression coefficients reveals that the fear of loss of power and inadequate information sharing subscales are significant positive predictors of quiet quitting behavior (*p* < 0.05). Conversely, the inadequate communication and lack of participative management subscales are not significant predictors (*p* > 0.05). Furthermore, the bivariate correlations between the mushroom management style subscales and quiet quitting behavior are positive, moderate, and statistically significant (0.32 ≤ bivariate *r* ≤ 0.42, *p* < 0.01). When partial correlations are examined, the highest correlation is observed between quiet quitting behavior and the fear of loss of power subscale (partial *r* = 0.11). Finally, based on the standardized beta coefficients (*β*), the predictors can be ordered by their relative importance on quiet quitting behavior as follows: fear of loss of power, inadequate information sharing, lack of participative management, and inadequate communication.

Table 4 presents the results of the multiple regression analysis examining the predictive effect of mushroom management style on work-related performance.

**Table 4:** Results of multiple regression analysis predicting work-related performance based on mushroom management style

Predictor Variables	Work-related performance						
	B	SE	β	t	p	Bivariate *r*	Partial *r*
Constant	0.886	0.083		10.616	0.00**		
Inadequate information sharing	0.098	0.044	0.157	2.194	0.03*	0.318	0.102
Fear of loss of power	0.009	0.043	0.016	0.204	0.84	0.292	0.010
Inadequate communication	0.042	0.032	0.074	1.289	0.20	0.248	0.060
Lack of participative management	0.075	0.036	0.151	2.101	0.04*	0.316	0.098

*R* = 0.347; *R*^*2*^ = 0.121; *F*_(4, 455)_ = 15.595; *p* < 0.01

As observed in Table 4, the overall regression model significantly accounts for approximately 12% of the variance in work-related performance (*R*^*2*^ = 0.121, *F*_(4, 455)_ = 15.595, *p* < 0.01). Examination of the regression coefficients reveals that the inadequate information sharing and lack of participative management subscales are significant positive predictors of work-related performance (*p* < 0.05). Conversely, the fear of loss of power and inadequate communication subscales are not significant predictors (*p* > 0.05). Furthermore, the bivariate correlations between the mushroom management style subscales and work-related performance are positive, weak to moderate, and statistically significant (0.25≤ bivariate *r* ≤ 0.32; *p* < 0.01). When partial correlations are examined, the highest correlation is observed between work-related performance and the inadequate information sharing subscale (partial *r* = 0.10). Finally, based on the standardized beta coefficients (*β*), the predictors can be ordered by their relative importance on work-related performance as follows: inadequate information sharing, lack of participative management, inadequate communication, and fear of loss of power.

Table 5 presents the results of the multiple regression analysis examining the predictive effect of mushroom management style on indifference towards school.

**Table 5:** Results of multiple regression analysis predicting indifference towards school based on mushroom management style

Predictor Variables	Indifference towards school						
	B	SE	β	t	p	Bivariate *r*	Partial *r*
Constant	0.821	0.100		8.232	0.00**		
Inadequate information sharing	0.056	0.053	0.070	1.058	0.29	0.415	0.050
Fear of loss of power	0.228	0.051	0.323	4.438	0.00**	0.486	0.204
Inadequate communication	0.032	0.039	0.045	0.840	0.40	0.345	0.039
Lack of participative management	0.072	0.043	0.111	1.671	0.10	0.427	0.078

*R* = 0.499; *R*^*2*^ = 0.249; *F*_(4, 455)_ = 37.677; *p* < 0.01

As observed in Table 5, the overall regression model significantly accounts for approximately 25% of the variance in indifference towards school (*R*^*2*^ = 0.249, *F*_(4, 455)_ = 37.677, *p* < 0.01). Examination of the regression coefficients reveals that the only fear of loss of power subscale is a significant positive predictor of indifference towards school (*p* < 0.01). Conversely, the inadequate information sharing, inadequate communication, and lack of participative management subscales are not significant predictors (*p* > 0.05). Furthermore, the bivariate correlations between the mushroom management style subscales and indifference towards school are positive, moderate, and statistically significant (0.35≤bivariate *r* ≤ 0.49; *p* < 0.01). When partial correlations are examined, the highest correlation is observed between indifference towards school and the fear of loss of power subscale (partial *r* = 0.20). Finally, based on the standardized beta coefficients (*β*), the predictors can be ordered by their relative importance on indifference towards school as follows: fear of loss of power, lack of participative management, inadequate information sharing, and inadequate communication.

Table 6 presents the results of the multiple regression analysis examining the predictive effect of mushroom management style on work-related depersonalization.

**Table 6:** Results of multiple regression analysis predicting work-related depersonalization based on mushroom management style

Predictor Variables	Work-related depersonalization						
	B	SE	β	t	p	Bivariate *r*	Partial *r*
Constant	1.250	0.146		8.553	0.00**		
Inadequate information sharing	0.128	0.078	0.121	1.646	0.10	0.246	0.077
Fear of loss of power	0.062	0.075	0.067	0.823	0.41	0.239	0.039
Inadequate communication	0.054	0.056	0.056	0.952	0.34	0.196	0.045
Lack of participative management	0.053	0.063	0.062	0.843	0.40	0.230	0.039

*R* = 0.266; *R*^*2*^ = 0.071; *F*_(4, 455)_ = 8.678; *p* < 0.01

As observed in Table 6, the overall regression model significantly accounts for approximately 7% of the variance in work-related depersonalization (*R*^*2*^ = 0.071, *F*_(4, 455)_ = 8.678, *p* < 0.01). Examination of the regression coefficients reveals that none of the mushroom management style subscales emerge as significant independent predictors of work-related depersonalization (*p* > 0.05). Furthermore, the bivariate correlations between the mushroom management style subscales and work-related depersonalization are positive, weak, and statistically significant (0.20≤bivariate *r* ≤ 0.25; *p* < 0.01). When partial correlations are examined, the highest correlation is observed between work-related depersonalization and the inadequate information sharing subscale (partial r = 0.08). Finally, based on the standardized beta coefficients (*β*), the predictors are ordered by their relative contribution to work-related depersonalization as follows: inadequate information sharing, fear of loss of power, lack of participative management, and inadequate communication.

## Discussion and conclusion

This study examined how mushroom management influences quiet quitting among teachers, drawing on Social Exchange and Psychological Contract theories. The positive relationship found between these two variables mirrors established patterns where negative workplace perceptions correlate with teacher withdrawal. Dilekçi et al. [53] and Boz [54] associate burnout and overwork with similar withdrawal processes; however, the data in this study finding intentional information restriction, which is part of mushroom management, as an independent organizational variable of this tendency. While the literature focuses on protective factors such as psychological well-being [55], job satisfaction [56], and transformational leadership [54], these findings identify the practice of managerial information withholding as a factor that weakens the principle of reciprocity in the psychological contract. Consequently, limitations in information sharing appear to weaken the reciprocal nature of the psychological contract; this dynamic may contribute to the decreased engagement observed in this study.

The findings demonstrate that low information sharing and feelings of exclusion in mushroom management style are observed alongside a weakening of reciprocity in teacher-school relationships, consistent with Social Change Theory [39]. These dynamics also reflect the increasing perception of psychological contract breach [42], a context associated with employee withdrawal. In this sense, quiet quitting can be interpreted as a passive reflection of the negative exchange processes described by these theories. Therefore, the positive relationship between mushroom management style and quiet quitting behavior is consistent with both the current theoretical framework and current research findings in the literature.

The study revealed that teachers’ general perceptions of the mushroom management style, along with their perceptions of “fear of loss of power” and “lack of participative management”, were at a low level, while their perceptions of “inadequate information sharing” and “inadequate communication” were at a medium level. These findings are consistent with the results reported by Alakaşlı et al. [57], where teachers’ perceptions of the mushroom management style wereobserved at a low level. While these scores might suggest a transition toward more transparent school leadership, the moderate levels of inadequate information sharing and inadequate communication warrant critical attention. Although literature emphasizes that teacher participation and transparent communication are vital for organizational trust and commitment [58–61], the data suggest a degree of ambiguity in these practices. While the results indicate that fear of loss of power and lack of participative management were not perceived as dominant factors, they suggest that existing communication channels in schools may need further improvement.

In subscale analyses, low scores for fear of loss of power and lack of participative management point to school environments where managers are open to sharing authority. This finding is consistent with contemporary educational paradigms that link participatory leadership with organizational commitment [60, 62]. On the other hand, moderate scores observed in the dimensions of inadequate information sharing and inadequate communication provide evidence of a difference between participatory practices and daily communication processes.

Communication gaps are discussed in the literature alongside exclusion and low organizational commitment [63]. Within the framework of Social Exchange Theory, limited information flow is related to the levels of support and reciprocity perceived by teachers. From the perspective of Psychological Contract Theory, a lack of transparency is defined as a contract breach; this is a process in which teachers align their efforts with the basic task boundaries in the contract. In this context, quiet quitting in schools is a form of implicit withdrawal observed alongside communication barriers rather than overt conflict. The findings point to moderately inadequate information sharing and communication processes, this reflects a need to develop transparent communication channels in school administrations.

Social Exchange Theory defines the teacher-administration relationship within a framework of reciprocity and trust [64]. Low scores on the mushroom management scale indicate a climate in which administrators in these school environments exhibit a supportive stance, free from information restrictions or exclusionary practices. This is consistent with literature findings that transparency and supportive leadership are associated with organizational trust [65–67]. From a Psychological Contract Theory perspective, the school structure involves a situation where risks of contract breaches, such as information withholding and exclusion from decision-making processes, are rare [68]. While literature highlights that participatory management is positively related to favorable employee attitudes [69], the present study suggests that the school environment largely meets teachers’ expectations for open communication and inclusion, thereby supporting the stability of these reciprocal relationships.

The findings indicate that teachers’ general quiet quitting behaviors, alongside the subscales of “work-related performance” and “indifference towards school”, were at a very low level, while “work-related depersonalization” remained low. These results align with research findings by Karaman Kepenekci et al. [15], Gılıç et al. [70], and Sabahat and Turhan [71], reinforcing the conclusion that teachers in the sampled population maintain high professional sensitivity and task commitment. While these findings do not align with the moderate levels of quiet quitting reported by Çabuk and Çelik [56], the data suggest that most teachers maintain a strong intrinsic commitment to the profession. It is possible that the divergent findings observed in other studies—such as the moderate levels reported by Çabuk and Çelik [56] or the qualitative observations of Konal Memiş and Tabancalı [11] regarding quiet quitting as a response to perceived devaluation and financial strain—stem from specific contextual pressures. The results indicate a moderately positive correlation between mushroom management style and teachers’ quiet quitting behavior. This finding parallels the literature that associates mushroom management with harmful organizational outcomes [22, 25]. Similar findings are observed in higher education institutions; studies on research assistants associate mushroom management with negative organizational outcomes. This approach has an effect that increases organizational cynicism and workplace loneliness; weakens commitment and job performance [21, 72, 73].

According to the research findings, mushroom management style explains approximately 20% of the variance in quiet quitting. This percentage is significant considering the complex nature of quiet quitting, which consists of individual, organizational, and professional factors [74]. Numerous variables, such as workload, burnout, and organizational justice [10, 75], contribute to teachers’ withdrawal behaviors. In this context, the observation that mushroom management alone can explain one-fifth of this variance is noteworthy in demonstrating the impact of administrative practices on teacher behavior.

The attitudes of the administration towards information sharing, communication, and participation processes are directly related to the psychological commitment of teachers [76]. In this context, limiting the flow of information and communication is linked to quiet quitting. These findings indicate that quiet quitting is not only a phenomenon based on individual motivation but also on leadership and communication practices in school administration [10]. Accordingly, management approaches based on transparent, participatory, and open communication are considered as an option to balance the tendency towards quiet quitting [23].

Looking at the regression analysis results regarding the prediction of quiet quitting by the subscales of mushroom management, it was found that the subscales of fear of loss of power and inadequate information sharing were significant predictors of quiet quitting (*p* < 0.05). In contrast, the subscales of inadequate communication and lack of participative management did not significantly predict quiet quitting (*p* > 0.05).

Administrative practices in the school environment can be interpreted differently by teachers. In educational settings, teachers may sometimes tend to view behaviors such as withholding information or protecting power as a negative and deliberate approach on the part of the administrator [77]. When administrators adopt a defensive stance and restrict information sharing, it can create a sense of exclusion among teachers. It could be argued that such an assessment may have a more weakening effect on the relational trust foundation. Conversely, communication deficiencies or limitations on participatory management in the school environment can be viewed as structural limitations imposed by the system rather than intentional exclusion. This distinction suggests that not every managerial deficiency leads to the same level of emotional detachment or quiet quitting. This view parallels the literature on psychological contract breaches. Morrison and Robinson [78] note that employees’ reactions to intentional information or resource restrictions by a manager can be more pronounced than their reactions to maladaptations arising from systemic or structural failures.

This study expands upon the theories of Social Change and Psychological Contract, offering a different perspective on employee turnover processes. While the literature generally associates psychological contract breaches with active negative consequences, examining quiet quitting within the context of mushroom management presents a new dynamic. In opaque environments where information asymmetry is used as a power tool by managers, teachers’ efforts to restrict their activities can be seen as an attempt to restore the disrupted social balance. Thus, quiet quitting emerges not as a passive failure of engagement, but as an active equity-restoration mechanism—a self-protective effort to recalibrate expectations within an asymmetrical power dynamic.

Finally, while this study focuses on the Turkish public education system, its implications align with the broader international literature on educational and organizational management. The Turkish public school system provides a robust context for this research due to its highly centralized structure and culturally embedded high power distance. In such hierarchical environments, managerial behaviors related to power retention and information control are often more observable. By indicating that teachers may resort to quiet quitting when faced with intentional administrative exclusion—even in a traditionally collectivist culture where institutional loyalty is emphasized—this study contributes to the broader understanding of psychological contract theory. Findings allow us to consider the withdrawal of voluntary efforts in hierarchical structures, where leaders prioritize information restriction over engagement processes, not as a local phenomenon but as a more general organizational dynamic.

### Practical implications for educational practice

The results of this study carry significant implications for educational practice, particularly in relation to school leadership and organizational functioning. First, while teachers’ perceptions of the mushroom management style are generally low, their perceptions of inadequate information sharing and communication are moderate, suggesting that even limited disruptions in transparency and interaction may correspond to significant organizational challenges. Accordingly, school administrators should prioritize establishing open, timely, and inclusive communication channels. Regular information meetings, participatory decision-making processes, and transparent information flow can help foster trust among teachers and mitigate the risk of withdrawal behaviors.

Second, the moderately positive correlation found between the mushroom management style and quiet quitting behavior underscores the critical relevance of leadership practices to teacher behavior. Although this study determined that quiet quitting behavior among teachers was generally low, the sustainability of this positive dynamic appears to be closely linked to managerial approaches. Therefore, school administrators adopting more collaborative and participatory leadership approaches, while avoiding information-hoarding behaviors, can help foster teachers’ sense of organizational belonging and participation.

Third, the findings indicating that teachers maintain their work performance and do not exhibit indifference towards school reflect a strong sense of professional commitment and ethical responsibility within educational organizations. However, relying solely on teachers’ intrinsic motivation and sense of responsibility may not be sustainable in the long term. Therefore, it is crucial for educational institutions to actively recognize teachers’ efforts by cultivating a supportive organizational climate and providing a fair and respectful working environment.

Fourth, the fact that this study revealed that mushroom management style explained 20% of the variance in quiet quitting behavior underscores the importance of interventions aimed at reducing such negative management approaches. In this context, it is recommended that in-service training programs for school administrators include effective communication, transparency, and trust-building skills. Furthermore, developing feedback mechanisms within the education system where teachers’ opinions can be regularly gathered will contribute to the early identification of potential communication problems.

Fifth, although this research was conducted among public school teachers in Türkiye, the significance of the findings may extend beyond this national context. Since the managerial processes addressed in the study—such as information withholding, communication deficiencies, and exclusion from decision-making—are widely recognized phenomena observable in many organizational contexts, testing these findings in different cultural and organizational environments could offer valuable contributions to the literature. Similarly, quiet quitting has recently emerged as a global phenomenon, prevalent across various sectors and cultural backgrounds. Therefore, rather than merely presenting country-specific results, this study hopes to draw attention to a potentially broader dynamic: the ways in which managerial information asymmetry may shape employee withdrawal behaviors.

Finally, the findings indicate that quiet quitting behavior in educational organizations often manifests not as a sudden and overt reaction, but rather as a subtle and gradual withdrawal process associated with communication gaps. Therefore, improving the quality of daily interactions in schools and cultivating relational trust emerge as vital strategies for sustaining teacher commitment and promoting organizational effectiveness.

### Limitations and suggestions for future research

The findings of this study should be evaluated in light of certain limitations. First, the cross-sectional design precludes definitive causal inferences regarding the relationships between the variables. While the theoretical framework posits that the mushroom management style exacerbates quiet quitting, alternative causal explanations cannot be ruled out. For instance, a reverse causality scenario is plausible: teachers who are already experiencing quiet quitting or work-related depersonalization may become psychologically withdrawn, thereby perceiving their administrators’ routine communication gaps more intensely as intentional mushroom management. Therefore, future research employing longitudinal or experimental designs is needed to establish temporal precedence and clarify the causal directionality of these relationships in a more robust way. Second, the study’s data were collected solely through self-report scales. Using different data sources (e.g., managerial evaluations, observational data, or multiple data collection methods) in future studies could enhance the reliability of the findings. Third, the research findings are limited to data obtained from teachers working in public schools in Türkiye. Therefore, direct generalizations of the results to education systems in different countries or to private school contexts should be done cautiously. Fourth, this study determined that the mushroom management style has limited power (20%) to explain quiet quitting behavior. This indicates that quiet quitting behavior has a multidimensional structure and is influenced by different variables. The fact that 80% of quiet quitting behavior is explained by other variables suggests that future research should include variables such as organizational justice, organizational trust, perceived organizational support, favoritism, mobbing, burnout, work alienation, job satisfaction, teacher patience and professional commitment in the model. Finally, given that quiet quitting is a relatively nascent phenomenon within educational contexts, and because this research adopted a strictly quantitative approach, subjective experiences of teachers remain underexplored. Subsequent qualitative or mixed-methods investigations are needed to further refine the conceptual clarity of quiet quitting, unpack its underlying dimensions, and provide a richer, more comprehensive understanding of teachers’ perceptions and feelings regarding mushroom management practices.

## Data Availability

The datasets generated and/or analyzed during the present study are available upon reasonable request from the corresponding author.
